# Evaluation of Remaining Dentine Thickness in Mandibular Incisors After Shaping With Varied Tapered Instruments: A Cone Beam Computed Tomography (CBCT) Comparative Study

**DOI:** 10.7759/cureus.109810

**Published:** 2026-05-28

**Authors:** Tracy El Feghaly, Pascale Salameh, Issam Khalil, Roula El Hachem

**Affiliations:** 1 Department of Endodontics, Saint Joseph University of Beirut, Beirut, LBN; 2 Department of Primary Care and Population Health, University of Nicosia Medical School, Nicosia, CYP; 3 Department of Public Health, Institut National de Santé Publique, d'Épidémiologie Clinique et de Toxicologie-Liban (INSPECT-LB), Beirut, LBN; 4 Department of Pharmacy, Lebanese University, Beirut, LBN; 5 School of Medicine, Lebanese American University, Beirut, LBN; 6 Cranio-Facial Research Laboratory, Biomaterials Unit, Saint Joseph University of Beirut, Beirut, LBN

**Keywords:** cbct, dentine thickness, mandibular incisors, shaping, taper

## Abstract

Aim: This study evaluated by cone beam computed tomography (CBCT) the impact of canal enlargement using ProGresShape (REKITA, Zouk Mikael, Lebanon) rotary files with 5% and 7% tapers on remaining dentine thickness (RDT) in mandibular incisors with different root canal configurations.

Methods: Twenty-four mandibular incisors with two root canal configurations (n=12) were selected and scanned using CBCT. Canal shaping was performed in two steps: first with ProGresShape up to #25.05, followed by #25.07. After each step, a new CBCT scan was taken. RDT was measured at the coronal, middle, and apical thirds of the root, considering both mesial and distal walls. Statistical analyses were performed using repeated measures analysis of variance (ANOVA) (p<0.05).

Results: A statistically significant difference in RDT was observed after preparation with 5% and 7% taper files (p<0.05). The 5% taper preserved more dentine than the 7%. In both groups, the number of root surfaces measuring less than 0.5 mm increased significantly after shaping, particularly in the apical third. The 25.07 file resulted in more slices under 0.5 mm compared to the 25.05 file. Overall, shaping led to a notable reduction in dentine, raising concerns about the risks of perforation and fracture, especially apically.

Conclusion: Instruments with a smaller taper (<5%) are preferable for shaping mandibular incisors to preserve dentine thickness. However, clinicians must ensure effective disinfection protocols. Future research should explore irrigation activation protocols in conservative approaches for mandibular incisors with different configurations.

## Introduction

Root canal morphology can vary significantly, even among a single type of tooth [1]. Therefore, comprehension of the root canal morphology is a fundamental prerequisite for a favourable treatment outcome as it may help clinicians adapt the treatment protocol to have a proper approach to the case [1]. In particular, mandibular incisors are recognised to exhibit a diversity of morphological root canal configurations and cross-sectional shapes [2,3]. Despite the prevailing notion that typical mandibular central and lateral incisors have a single root canal (Vertucci type I), multiple studies found that the occurrence of a second canal ranged between 0.3% [4] and 48.1% [5]. The most prevalent type of two canals was Vertucci type III, where the dentinal bridge separates the root into two different canals and then merges back into a single canal at the apical level [6]. This variation concerning the incidence of the second canal in mandibular incisors can be linked to different ethnic backgrounds [7], ages [8], genders [9], and differences in research methodologies [10].

Excessive removal of dentine from the root wall during instrumentation may compromise the long-term prognosis of the tooth by increasing the risk of vertical root fracture (VRF) or strip perforation [11] that could lead to extraction. The proportion of preserved dentine is essential to improve the long-term survival of endodontically treated teeth [12]. Building upon this concept, the philosophy of minimally invasive endodontics (MIE) gained recognition over the last two decades; it has emerged as a promising approach in reducing iatrogenic damage and ensuring a higher survival rate of root canal-treated teeth [12]. Yet, the ability to achieve an equilibrium between the necessary apical enlargement for an optimal disinfection of the root canal system and the required dentine thickness to withstand mechanical stress has always been a longstanding controversial topic [13]. Recent studies claim that using a small and regressive tapered instrument such as 20/0.4 and 25/0.4 combined with ultrasonically activated irrigant is equally efficient in achieving canal cleanliness when compared to largely prepared canals [14,15].

Various techniques have been described to evaluate dentine thickness in the radicular tooth walls, such as serial sectioning [16], conventional radiography (CR) [17], micro-computed tomography (micro-CT) [18], and cone beam computed tomography (CBCT) [19]. With the introduction of CBCT, it has been possible to assess dentine thickness in a non-invasive way before and after root canal instrumentation [20]. Raiden et al. found that CR is not an accurate tool to measure residual dentine thickness [17]. On the other hand, Xu et al. demonstrated a strong correlation between measurements carried out by micro-CT and those carried out by CBCT [21]. 

The literature points out numerous studies reporting the variation in morphological features present in mandibular incisors; however, there is no clear evidence on the ideal approach to prepare these teeth while maintaining a dentine thickness without excessive wear. Therefore, this ex vivo investigation aimed to evaluate, using CBCT analysis, remaining dentine thickness (RDT) after shaping progressively the root canal with 5% and 7% tapered instruments in mandibular incisors with different root canal configurations. The null hypothesis tested was that shaping the root canal using different rotary instruments with varying tapers of 5% and 7% does not significantly impact the amount of removed root dentine.

## Materials and methods

Sample size calculation and selection 

After obtaining approval from the Research Ethics Committee of Saint Joseph University of Beirut (approval number: Tfemd/2024/42), this ex vivo study was conducted at the Dental Care Center of Saint Joseph University in Beirut, Lebanon. To determine the sample size, a power analysis for a repeated measures analysis of variance (ANOVA) (within-subjects factor) was conducted using G*Power software 3.1.9.7 for Windows (Heinrich-Heine-Universität Düsseldorf, Düsseldorf, Germany). A power of 0.95, an alpha level of 0.05, and three measurements were considered, and an effect size of 0.5 was calculated based on a previous study [22]. The minimum sample size required is 12 teeth in total. 

Specimen selection and imaging 

Fifty permanent mandibular incisors extracted for unrelated reasons for this study were scanned by a CBCT device (Axeos, Dentsply Sirona, Charlotte, NC, USA) taken at 90 kV and 12 mA with a voxel size of 0.04 mm. The demographic factors, including sex, age, and ethnicity, of the patients from whom the teeth were extracted were not specified. Canal configuration was analysed, resulting in the selection of 24 mandibular incisors for this study. These were equally divided into incisors presenting Vertucci type I configuration (n=12) and incisors presenting Vertucci type III configuration (n=12). 

All of the samples met the following criteria: absence of caries, fully formed apices, absence of external or internal root resorption, non-calcified root canals, and no previous endodontic treatment. A digital periapical radiograph was taken in the buccolingual direction to determine whether it was a single root canal or two canals. All teeth presented similar root lengths of 12 mm, to minimise the influence of shape variability on the results. In all Vertucci type III teeth examined, a single canal was present in the coronal third, which then splits into two separate canals in the middle third before merging back into a single canal in the apical third. Specifically, at 11 mm from the apical foramen (AF), there was one canal; at 8 mm from the AF, which corresponds to the middle third, two canals were present; and at 4 mm from the AF, the canals had already merged back into a single canal. The selected teeth were immersed in 5.25% sodium hypochlorite (NaOCl) for 24 hours before cleaning them of calculus and plaque with an ultrasonic scaler. 

Root canal preparation 

A conventional access cavity was prepared, and the apical patency was established with 10 K-files (Dentsply Sirona, Ballaigues, Switzerland), and the working length (WL) was set at 0.5 mm short from the AF. The glide path was performed with the ProGresShape PreShape instrument 12.03/05/07 (ProGresShape, REKITA, Zouk Mikael, Lebanon). Hot glue was applied at the apical third of the root of every tooth which was placed into wax to grant mechanical stability throughout the experiment. The canal preparation was performed sequentially in three steps using in-and-out motion with the ProGresShape rotary file system (REKITA), adapted to the endodontic motor (X-Smart; Dentsply Sirona, Charlotte, NC, USA) set at 400 rpm and 2 Ncm. 

The first step involved enlarging the canal with files in the following order: ProGresShape 18.05 and ProGresShape 25.05. The second step consisted the preparation until ProGresShape 25.07. Following every three in-and-out motion strokes, the shaping instrument was retrieved from the canal and was carefully cleaned with a wet gauze. After achieving the access opening and the glide path step, each canal was irrigated with 2 cc of 2.5% NaOCl using a 31-G needle attached to a 3 mL disposable syringe. The same was carried out after the removal of each instrument from the canal along with the recapitulation using a 10 K-file. A single operator, specialist in Endodontics, performed the entire procedure to reduce inter-operator variability. After the completion of every step, a new CBCT scan was taken. 

Image analysis 

Three CBCT scans were taken per tooth: before the endodontic procedure and after shaping with 25.05 and 25.07 instruments. To ensure reproducible positioning across all imaging sessions, each tooth was placed in the same custom-made mold for every CBCT acquisition, maintaining a consistent orientation and minimising positional variability between scans. The collected data was imported as a Digital Imaging and Communications in Medicine (DICOM) file on ITK-SNAP software version 4.0.2 (free software under the GNU General Public License developed by the National Institutes of Health, the US National Institute of Biomedical Imaging and BioEngineering, the US National Library of Medicine, the Universities of Pennsylvania and North Carolina, and an independent developer group) for superimposition and then on 3D slicer software version 5.6.1 (Slicer Community in collaboration with Brigham and Women's Hospital, Harvard Medical School, Boston, MA, USA) for image analysis and sectioning. After superimposition, three axial sections were performed for each tooth's CBCT image: at 11 mm, at 8 mm, and at 4 mm from the AF. For each section, two measurements of the remaining dentine were carried out per canal, one for the mesial and distal walls, considering the distance from the canal lumen to the external surface of each proximal wall.

All measurements were carried out in millimeters and were presented as means. To differentiate segments after each step, the same tooth was color-coded using the "display" module in 3D slicer: yellow for the preoperative image, cyan for post-25.05 shaping, and red for post-25.07 shaping. A single examiner performed all the measurements to ensure uniformity in the assessment process. 

Statistical analysis 

Data was analysed using IBM SPSS Statistics for Windows, V. 26.0 (IBM Corp., Armonk, NY, USA). Descriptive statistics included means (standard deviation) for continuous variables. A repeated measures ANOVA was performed, aiming to compare the mean dentine thickness measurements of preoperative, post-25.05, and post-25.07 enlargements. Statistical significance was set at p<0.05.

## Results

**Figure 1:**
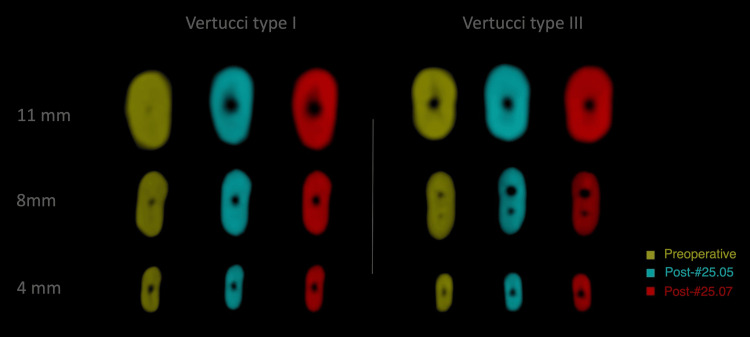
Axial cross-sectional views of Vertucci type I and Vertucci type III mandibular incisors, preoperative (yellow), post-#25.05 (blue), and post-#25.07 (red), at three different levels from the apical foramen

In Vertucci type I incisors, a statistically significant difference was observed in the RDT between #25.05 and #25.07 preparations at three different levels: 11 mm, 8 mm, and 4 mm from the AF (Figure 1, Table 1). For the mesial section, at 11 mm, the mean RDT was significantly higher for #25.05 (1.04 mm) compared to #25.07 (0.93 mm) (p<0.001), with no slices measuring less than 0.5 mm. Similarly, at 8 mm, the mean RDT was 0.84 mm for #25.05 versus 0.76 mm for #25.07 (p<0.001), again with no slices measuring less than 0.5 mm. At 4 mm, the mean RDT was 0.61 mm for #25.05 compared to 0.51 mm for #25.07 (p<0.001). At this level, the number of slices with an RDT below 0.5 mm increased slightly across steps, from 1 (preoperative) to 3 (post-#25.05) and 5 (post-#25.07). For the distal section, the mean RDT was also significantly higher for #25.05 across the same levels: at 11 mm, 1.04 mm versus 0.94 mm (p<0.001); at 8 mm, 0.83 mm versus 0.76 mm (p<0.001); and at 4 mm, 0.52 mm versus 0.45 mm (p<0.001). No slices measured less than 0.5 mm at 11 mm or 8 mm from the AF, while at 4 mm, the number of slices below this threshold increased from 0 (preoperative) to 5 (post-#25.05) and 9 (post-#25.07). 

**Table 1:** Minimal mean dentine thickness (mm)±standard deviation of proximal walls in Vertucci type I incisors measured at 11 mm, 8 mm, and 4 mm from the apical foramen preoperatively and after the #25.05 and #25.07 preparations, including the number of slices measuring less than 0.5 mm D: distal wall; M: mesial wall

		Preoperative	Post-#25.05	Post-#25.07	P-value	F-value
M	11 mm	1.26±0.11 (0/12)	1.04±0.12 (0/12)	0.93±0.11 (0/12)	<0.001	3.64
	8 mm	0.99±0.11 (0/12)	0.84±0.12 (0/12)	0.76±0.11 (0/12)	<0.001	163
	4 mm	0.68±0.13 (1/12)	0.61±0.14 (3/12)	0.51±0.14 (5/12)	<0.001	75.36
D	11 mm	1.24±0.06 (0/12)	1.04±0.06 (0/12)	0.94±0.07 (0/12)	<0.001	138.62
	8 mm	0.98±0.09 (0/12)	0.83±0.11 (0/12)	0.76±0.11 (0/12)	0.001	67.9
	4 mm	0.60±0.08 (0/12)	0.52±0.09 (5/12)	0.45±0.08 (9/12)	<0.001	71.26

In all Vertucci type III incisors, a statistically significant difference in RDT was found between #25.05 and #25.07 preparations, using the preoperative group as a reference at three levels: 11 mm, 8 mm, and 4 mm from the AF (Figure 1, Table 2). A significantly higher mean RDT was observed for the mesial wall at 11 mm from the AF, with values of 1.06 mm for #25.05 and 0.99 mm for #25.07 (p<0.001), with no slices exhibiting an RDT below 0.5 mm at this level. At 8 mm, the RDT for the buccal canal was 0.85 mm for #25.05 versus 0.78 mm for #25.07 (p<0.001), and for the lingual canal at the same level, the RDT was 0.84 mm for #25.05 compared to 0.79 mm for #25.07 (p<0.001), both without slices below 0.5 mm. At 4 mm from the AF, the RDT was 0.61 mm for #25.05 compared to 0.56 mm for #25.07 (p<0.001), with the number of slices below 0.5 mm increasing from 0 (preoperative) to 1 (post-#25.05) and 3 (post-#25.07). For the distal wall, a statistically significant difference in RDT between #25.05 and #25.07 preparations was also found at the same three levels. At 11 mm from the AF, the RDT for the distal wall was 1.05 mm for #25.05 compared to 0.91 mm for #25.07 (p<0.001), with no slices measuring less than 0.5 mm. At 8 mm, for the buccal canal, the values were 0.78 mm for #25.05 and 0.70 mm for #25.07 (p<0.001). The number of slices below 0.5 mm increased slightly from 0 (preoperative) to 1 (post-#25.05), remaining 1 (post-#25.07). For the lingual canal at the same level, the RDT was 0.78 mm for #25.05 compared to 0.72 mm for #25.07 (p<0.001), with no slices measuring less than 0.5 mm. At 4 mm from the AF, the RDT was 0.51 mm for #25.05 versus 0.46 mm for #25.07 (p<0.001). The number of slices measuring less than 0.5 mm increased from 1 (preoperative) to 6 (post-#25.05) and 7 (post-#25.07).

**Table 2:** Minimal mean dentine thickness (mm)±standard deviation of proximal walls in Vertucci type III incisors measured at 11 mm, 8 mm, and 4 mm from the apical foramen preoperatively and after the #25.05 and #25.07 preparations, including the number of slices measuring less than 0.5 mm D: distal wall; M: mesial wall; BC: buccal canal; LC: lingual canal

		Preoperative	Post-#25.05	Post-#25.07	P-value	F-value
M	11 mm	1.26±0.13 (0/12)	1.06±0.14 (0/12)	0.99±0.13 (0/12)	<0.001	121.97
	8 mm BC	1.02±0.09 (0/12)	0.85±0.11 (0/12)	0.78±0.13 (0/12)	<0.001	35.91
	8 mm LC	1.06±0.07 (0/12)	0.84±0.08 (0/12)	0.79±0.08 (0/12)	<0.001	72.4
	4 mm	0.69±0.11 (0/12)	0.61±0.12 (1/12)	0.56±0.12 (3/12)	<0.001	35.43
D	11 mm	1.23±0.16 (0/12)	1.05±0.18 (0/12)	0.91±0.09 (0/12)	<0.001	23.17
	8 mm BC	0.97±0.09 (0/12)	0.78±0.09 (1/12)	0.70±0.06 (1/12)	<0.001	76.6
	8 mm LC	0.94±0.06 (0/12)	0.78±0.10 (0/12)	0.72±0.11 (0/12)	<0.001	53.11
	4 mm	0.60±0.10 (1/12)	0.51±0.09 (6/12)	0.46±0.09 (7/12)	<0.001	52.82

## Discussion

This study aimed to assess the amount of RDT using CBCT at three canal levels after sequentially shaping with a 5% tapered instrument, followed by a 7% tapered instrument, in mandibular incisors with two root canal configurations. RDT was measured after each step to determine the impact of each instrument on root thickness. 

The morphology of mandibular incisors has received considerable attention in scientific literature, elucidating their diverse configurations and prevalence based on various demographic parameters [4,6,23,24]. Some studies highlight the predominance of Vertucci type I configuration as the most prevalent among mandibular incisors, followed by Vertucci type III configuration, which was also considerable on a global scale [25]. Furthermore, investigations into the cross-sectional morphology of these teeth indicate thin mesio-distal dimensions in comparison with their bucco-lingual ones [26,27]. According to Katz and Tamse, the distal wall is generally thinner than the mesial wall [26]. This finding was also supported by our results. Additionally, mandibular incisors present invagination at both proximal sides, with a noticeable accentuation observed on the distal aspect, contributing to lower mean dentine thickness in the distal section [28]. This anatomical particularity poses challenges, particularly during the mechanical preparation of the root canal, where clinicians risk over-instrumentation of proximal walls, prompting our investigation into mesial and distal dentine thickness. Despite the importance of these observations, there is still a significant lack of scientific evidence regarding the safety and effectiveness of mechanical enlargement within this type of teeth. 

The selection of CBCT as an imaging modality to assess dentine thickness emerges from several considerations. It is a non-destructive modality ensuring the preservation of tooth integrity [29]. Moreover, Xu et al. found a high correlation between dentine thickness measurements carried out through CBCT and those derived from micro-CT, a method known for its accuracy but limited by its expense, time requirements, and occasional unavailability [21,29]. Thus, CBCT proves to be a practical choice, balancing between accuracy and accessibility for our study's needs. To ensure the validity of our findings, we used two different tapers from the same rotary system with an identical tip diameter (ProGresShape #25), eliminating potential interference of factors such as file design and metallurgical properties that could affect the cutting ability of the instrument. Additionally, the root canal preparation was performed with 5% and 7% tapers in two steps within each canal to avoid the confounding factors of anatomical variation across different roots. 

Overall, our results indicated a statistically significant difference regarding RDT between the 5% and 7% shaping protocols for all mandibular incisors at the three canal thirds (Table 1 and Table 2). Thus, the null hypothesis was rejected. 

Based on the results found by Zhou et al., the RDT in danger zones should not decrease to more than 0.5 mm to prevent perforation risk [30]. Furthermore, Elayouti et al. stated that 0.5 mm is chosen as a standardized value in studies assessing the RDT because in narrow roots it represents the removal of more than 50% of the original dentine [31]. In our study, among the 12 teeth with a single canal, 11 exhibited at least one proximal wall measuring less than 0.5 mm, at 4 mm from the AF, after instrumentation with a 25.07 file, while only seven teeth displayed at least one proximal section measuring less than 0.5 mm after instrumentation with a 25.05 file. As for the teeth with two canals, the results revealed minor differences; out of the 12 teeth, nine displayed at least one proximal wall measuring less than 0.5 mm at the apical level after enlargement with the 25.07 file. Similarly, six teeth exhibited at least one proximal portion measuring less than 0.5 mm after enlargement with the 25.05 file. These findings indicate that enlarging the canal with the 25.07 file in mandibular incisors led to a higher incidence of dentine walls measuring <0.5 mm compared to the 25.05 file. However, it should be noted that even with the 25.05 file, a considerable number of teeth presented at least one proximal wall measuring less than 0.5 mm. 

It has been demonstrated that a higher enlargement of the root canal results in a higher bacterial reduction [32]. Specifically, in the case of necrosis, increasing the canal taper is useful as it increases the irrigant flow and the reduction of bacterial load [33]. On the other hand, increasing the taper results in an additional reduction of dentine from the coronal and the middle thirds; this may leave the tooth less resistant to stresses, thereby risking complications such as VRF [34]. Practitioners must be cautious in selecting appropriate instruments to achieve an equilibrium between achieving adequate disinfection and preserving dentine thickness to prevent complications. Yared and Ramli demonstrated that effective canal cleanliness can be achieved even in uninstrumented canals, by ultrasonic agitation and intracanal heating [35]. Further investigations in this regard are needed in case of mandibular incisors with various canal configurations following less invasive canal enlargement protocols than 25.05. 

However, this study is limited by the unprecise age of the teeth, which can influence the dentine thickness due to the deposition of dentine resulting from physiological and pathological processes. Furthermore, the use of CBCT may result in an underestimation of dentine thickness compared to micro-CT [21]. Therefore, it is recommended to conduct another study using micro-CT for a more accurate assessment of dentine thickness, along with a larger sample size to better understand the implications of root canal shaping on root integrity. 

## Conclusions

Within the limitations of this study, the findings indicate that canal shaping with a 5% taper is significantly less invasive than with a 7% taper. However, both tapers can cause substantial dentine reduction, potentially compromising the structural integrity of the root which may increase the risk of perforation or VRF, particularly in the apical third. Clinicians are therefore advised to adopt conservative shaping protocols that preserve dentine while maintaining effective root canal disinfection. Further research is necessary to validate these findings and to develop safer and more efficient shaping strategies, with particular emphasis on irrigation activation techniques in the conservative treatment of mandibular incisors with varying canal configurations.
